# The Novel Use of the Lateral Scanogram to Detect Cruciate Deficiency in Children With Congenital Femoral Deficiency

**DOI:** 10.7759/cureus.40799

**Published:** 2023-06-22

**Authors:** Tristen N Taylor, Callie S Bridges, Benjamin M Martin, Scott McKay, Jaclyn F Hill

**Affiliations:** 1 Department of Orthopedic Surgery, Texas Children's Hospital, Houston, USA

**Keywords:** cruciate deficiency, anterior tibial translation, proximal femoral focal deficiency, congenital femoral deficiency, lateral scanogram

## Abstract

Congenital femoral deficiency (CFD) is often associated with cruciate ligament deficiency. The lateral scanogram may be a potential solution to some limitations for detecting instability associated with cruciate ligament deficiency. This qualitative case study identified two children with congenital femoral deficiency who were assessed with a lateral scanogram and had their results correlated to the clinical examination and MRI. Both cases identified a child with congenital femoral deficiency, one with a total leg length discrepancy (LLD) of 12 cm and the next with 6.5 cm. The weight-bearing lateral scanogram revealed anterior tibial translation, indicating knee instability. Both patients will undergo anterior cruciate ligament (ACL) reconstruction prior to limb lengthening. The lateral scanogram is a useful imaging modality that is capable of detecting anterior tibial translation, and thereby knee instability, in children with congenital femoral deficiency. Larger studies utilizing and evaluating the benefits of lateral scanograms are warranted.

## Introduction

Congenital femoral deficiency (CFD), a spectrum of pathology that encompasses congenital short femur and proximal femoral focal deficiency (PFFD), is a rare congenital anomaly with an incidence of 1.1-2.0 in 100,000 live births worldwide [1-4] and a male-to-female ratio of 1:2 [2]. A single underlying genetic cause for CFD has not been elucidated, and most available data points to the teratogenic or idiopathic somatic mutations of cells within the developing limb bud versus a hereditary etiology [5,6].

A deficiency of the cruciate ligaments is commonly associated with CFD and other various congenital longitudinal deficiencies of the lower limb [7-10]. A systematic review of CFD and associated ligament deficiencies in the knee noted that there is not enough support in the literature to back the routine reconstruction of the cruciate ligaments in patients with CFD [4]. In higher-functioning individuals, congenital absence is often well tolerated [10]. However, in children undergoing limb lengthening with severe CFD, anteroposterior knee subluxation occurs frequently and may often be symptomatic [4,10-12].

Despite the association between CFD and cruciate ligament deficiency, routine screening and treatment for these patients vary among practice, especially in patients undergoing limb lengthenings [4,7,11,13]. While anterior scanograms, or 2-3 radiographs of the lower extremities in the anteroposterior plane stitched together, are used to assess leg length discrepancy (LLD), the use of a scanogram from the lateral view has not yet been described as a method for screening in CFD patients suspected to have cruciate ligament deficiencies. Our case report describes two patients with CFD that were found to be cruciate ligament-deficient on physical examination and by lateral scanogram. Our goal is to describe how lateral scanograms may be used to effectively assess instability associated with cruciate ligament deficiency in patients with CFD.

## Case presentation

Case 1

**Figure 1:**
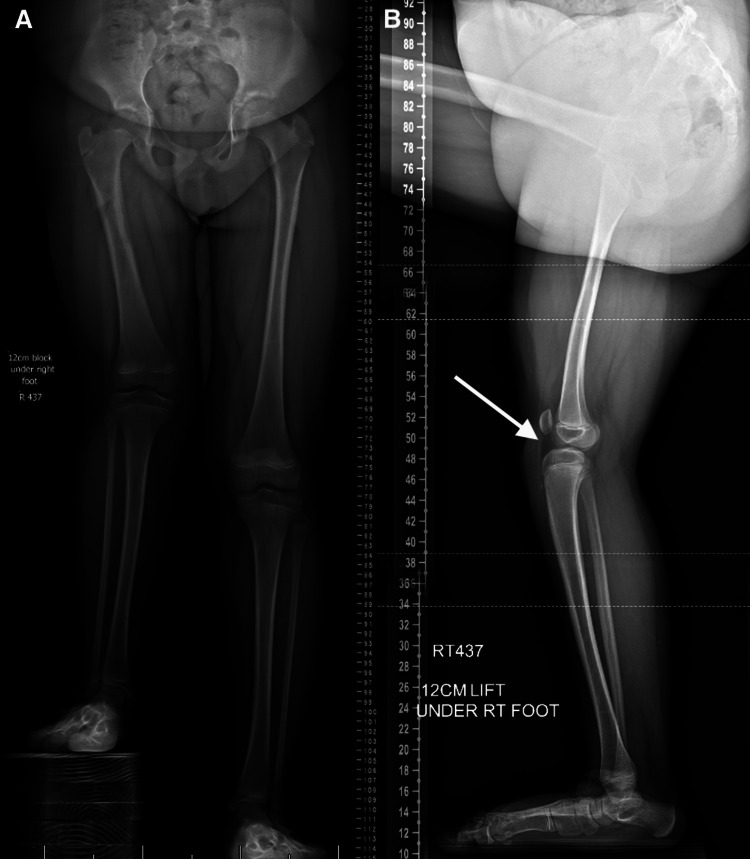
Anterior and Lateral Scanograms of Patient 1 (A) Anterior scanogram depicting 12 cm leg length difference of the right leg. (B) Lateral scanogram showing anterior tibial translation (white arrow)

A 10-year-old female with a history of right congenital femoral deficiency and valgus deformity presented for limb lengthening (Figure 1). She currently wears a shoe lift of 5.5 cm and complains of pain after walking and occasional instability in her knee.

On physical examination, the patient walked with a short-legged gait with genu valgum. With a 12 cm block under her right foot, her pelvis was level and spine straight on Adam's forward bend test. She had no end point on the anterior drawer test.

**Figure 2:**
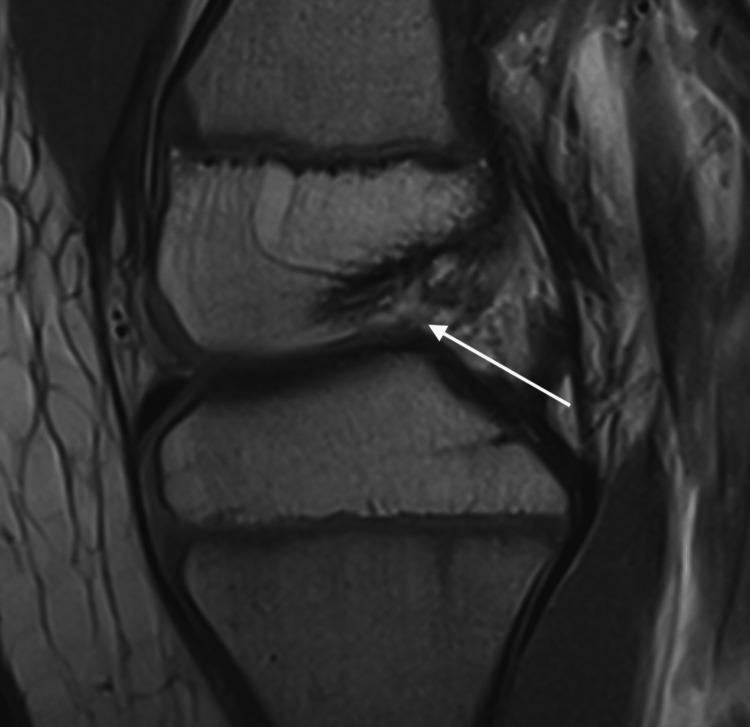
Lateral Right Knee MRI of Patient 1 White arrow: the absence of anterior cruciate ligament

Anterior scanogram revealed a right femoral length of 28.6 cm versus left femoral length of 40.2 cm and right tibia length of 31.2 cm versus left tibia length of 31.8 cm. The lateral scanogram revealed an anteriorly translated tibia with a posterior tibial slope of 15 degrees. A follow-up MRI revealed anterior cruciate ligament (ACL) deficiency and a posterior cruciate ligament (PCL) with decreased ligament caliber (Figure 2).

After discussion with family, the patient was scheduled to undergo a combined intra-articular and extra-articular ACL reconstruction and extra-articular PCL reconstruction with the ipsilateral iliotibial band, right distal medial femoral hemi-epiphysiodesis to address her knee valgus, and contralateral epiphysiodesis. The plan to address her leg length discrepancy is two femoral lengthenings with internal lengthening nail.

Case 2

**Figure 3:**
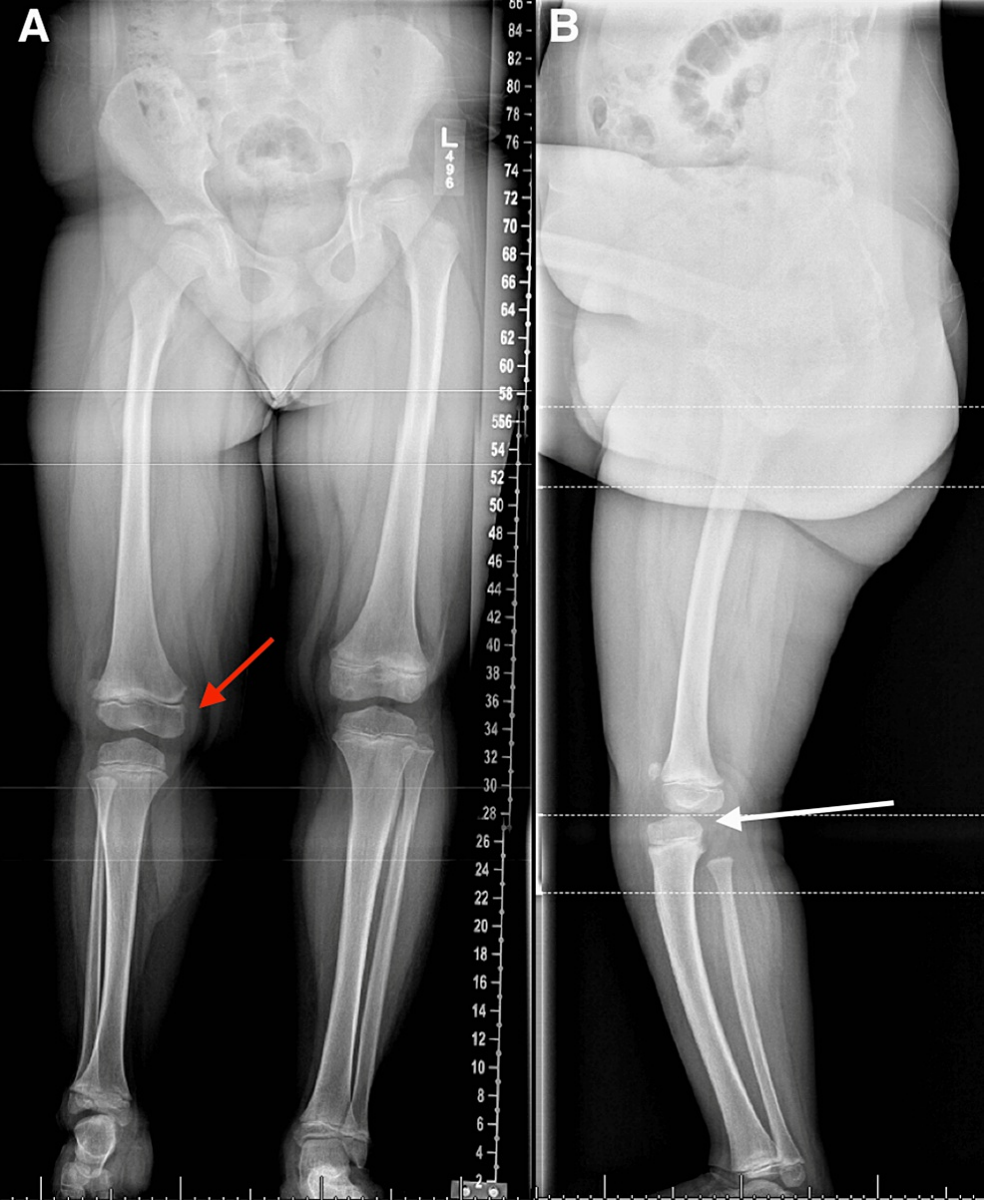
Anterior and Lateral Scanograms of Patient 2 (A) Anterior scanogram depicting leg length discrepancy of 6.5 cm (red arrow). (B) Lateral scanogram showing anterior tibial translation (white arrow) indicating cruciate ligament deficiency

A six-year-old male presented with his father for LLD (Figure 3). He had a history of right clubfoot with only four toes that was treated with serial casting and tenotomy. His LLD has been managed with orthotics and currently has an external lift on the right shoe.

On physical examination, he ambulated unassisted and had wide symmetric hip abduction, right genu valgum, and positive Galeazzi with the right femur 1.5 cm shorter than the left. His right tibia appeared 5 cm shorter than the left. His right foot only had four rays but otherwise normal dorsiflexion and plantarflexion. His pelvis was level with a 6.5 cm block under his right foot. His right knee demonstrated no end point on anterior drawer.

**Figure 4:**
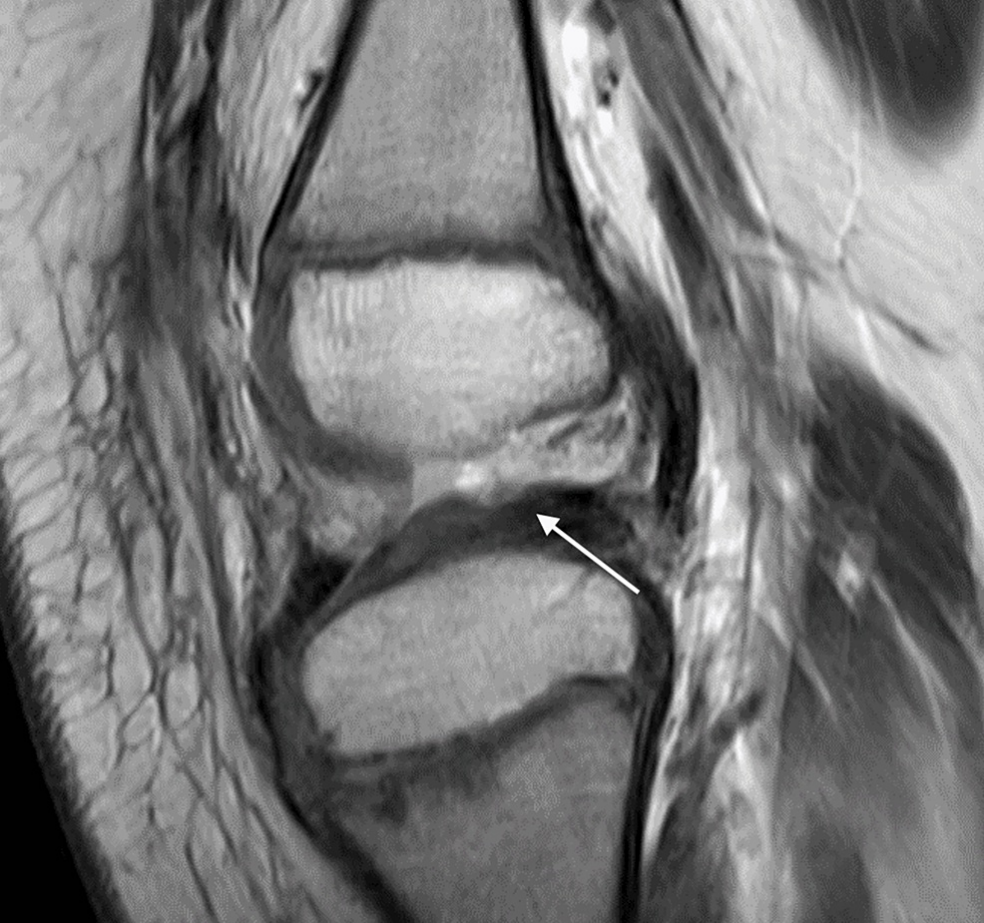
Lateral View of Right Knee MRI of Patient 2 Note the absence of the anterior cruciate ligament (white arrow) and anterior tibial translation

Anterior scanogram revealed a right femoral length of 36.1 cm versus left femoral length of 37.2 cm and right tibia length of 26.8 cm versus left tibia length of 31.2 cm. His lateral scanogram revealed the anterior translation of the tibia with the knee in extension. A follow-up MRI revealed the absence of the ACL and PCL (Figure 4).

This patient was diagnosed with right fibular hemimelia, right CFD, right genu valgum, ACL and PCL deficiency with knee subluxation, and an LLD of 6.5 cm (the projected LLD was 9.3 cm). The patient underwent hemi-epiphysiodesis of the medial distal femur and extra-articular and intra-articular reconstruction of the ACL and extra-articular PCL construction in preparation for limb-lengthening surgery. Leg length discrepancy will be addressed with the future lengthening of his tibia and possibly femur.

## Discussion

Cruciate ligament deficiency in patients with CFD undergoing lengthening is important to identify for surgical planning. Recurrent swelling and instability have previously been reported in patients with congenital aplasia of the ACL [9,14], and subluxation or dislocation during lengthenings also may occur in patients with CFD [9,11,15]. Torzilli et al. described in cadaveric models how the knees with a sectioned ACL displayed a "substantial anterior neutral-position shift" of the tibia during loading compared to native knees [16]. This was corroborated by Beynnon et al. who radiographed living knees with torn ACLs versus healthy knees, with and without loading, and found a fourfold increase in anterior translation [17]. They posed this increased laxity in the sagittal axis as a potential risk for meniscal and osteochondral damage associated with cruciate deficiency. Furthermore, the risk of severe early knee arthritis may be increased during the limb-lengthening process in patients with CFD and preoperative knee instability, due to abnormal pressure on articular cartilage, resulting in degeneration [18]. This was corroborated by Jeong et al., who reported an arthritis rate of 78% in CFD patients after lengthenings and a trend toward a positive correlation between preoperative knee instability and degenerative arthritis at the last follow-up [11].

**Figure 5:**
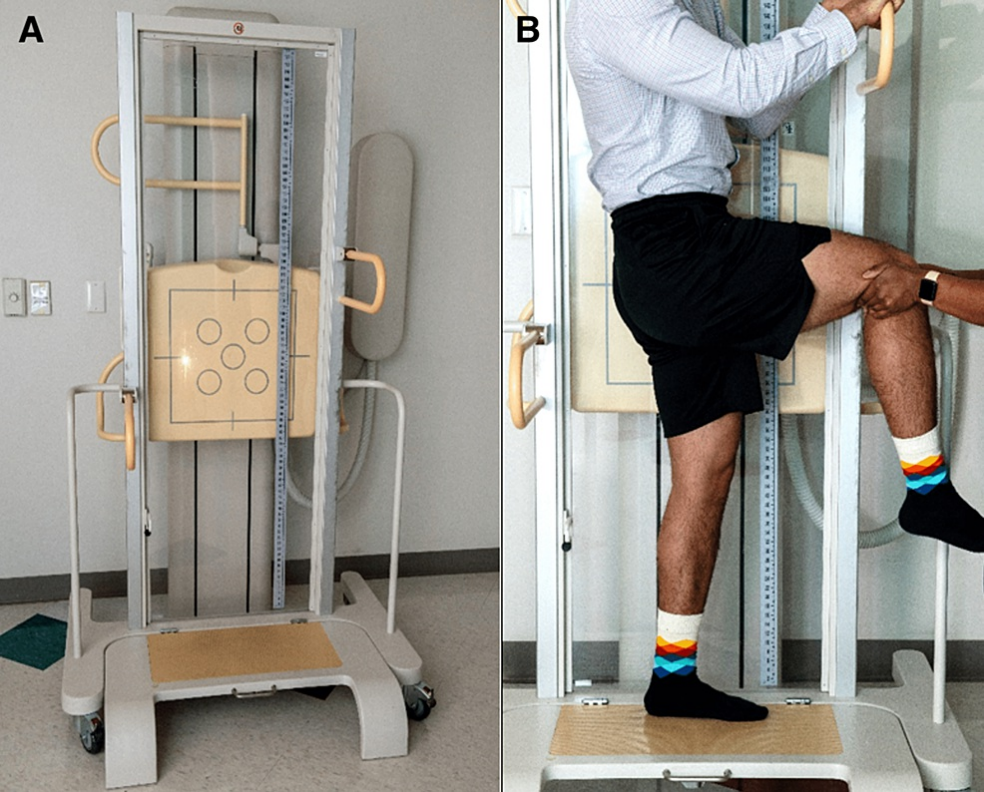
Proper Positioning for Lateral Scanogram (A) The scanogram apparatus that the patients stand in for anterior and lateral scanograms. (B) Proper positioning for a lateral scanogram. Note that the anterior-most hip (right leg) is flexed to 90 degrees and may be supported at the knee. Although an adult is used for modeling in this figure, the proper positioning of all patients regardless of age, BMI, or gender should remain consistent with what is displayed. Image credits: X Author

Due to these potential consequences, children with CFD are routinely evaluated with anterior and lateral scanograms in our practice. After the anterior scanogram is obtained, the lateral view can be taken accordingly (Figure 5). The patient is radiographed at a source image receptor distance of 102 cm. The patient should stand upright facing laterally such that their index limb is positioned distal to the tube and their femoral condyles parallel to the radiograph's beam. The handlebars of the scanogram apparatus should be adjusted to chest height for the patient to grab for stability, and the anterior-most hip should then be flexed to 90 degrees. Depending on habitus or strength, this limb may need to be stabilized by a stool or radiology technician to prevent leaning too much on the index limb.

The majority of ACL deficiencies can be diagnosed through history and clinical examination. The anterior drawer, Lachman, and pivot shift test are commonly used to detect clinically the absence or tear of an ACL. For surgical planning, the confirmation of knee instability, and the detection of concomitant bony abnormalities, correlation to imaging is necessary. The lateral scanogram is helpful to obtain compared to standard radiographs of the knee because it allows for the visualization of the entire lower limb in two planes while weight-bearing. Standard lateral radiographs provide the visualization of the bony anatomy from an orthogonal view to the anterior scanogram, but lateral scanograms may be quickly obtained after the anterior scanogram while also providing views of the proximal femur and distal tibia, which may be the pathological areas of interest during treatment. In contrast to routine screening with MRIs, which can cost on average 3,300 USD [19], the weight-bearing aspect of the lateral scanogram provides insight to how the knee behaves during their gait, and the distinct anterior translation of the tibia provides evidence of cruciate deficiency and instability. Furthermore, many patients with CFD require routine screening for their LLD by anterior scanogram; thus, a rotation in stance for the lateral scanogram is a prudent and efficient method for detecting concomitant instability.

## Conclusions

CFD is commonly associated with cruciate ligament deficiency that can result in an unstable knee. Efficient screening for cruciate deficiencies in these patients is important to ensure proper care and prevent potential adverse outcomes of any planned lengthening surgeries. We reported two cases of patients with CFD who were found to have a cruciate deficiency on lateral scanogram by anterior tibial translation. To our knowledge, this is the only study that reports on the use of lateral scanograms in CFD patients. Lateral scanograms are an effective method to evaluate for bone or ligamentous deformity/deficiency in patients with CFD.
